# Resistance to KRAS inhibition in advanced non-small cell lung cancer

**DOI:** 10.3389/fonc.2024.1357898

**Published:** 2024-05-23

**Authors:** Katherina Bernadette Sreter, Maria Joana Catarata, Maximilian von Laffert, Armin Frille

**Affiliations:** ^1^ Department of Pulmonology, University Hospital Centre “Sestre Milosrdnice”, Zagreb, Croatia; ^2^ Pulmonology Department, Hospital de Braga, Braga, Portugal; ^3^ Tumour & Microenvironment Interactions Group, I3S-Institute for Health Research & Innovation, University of Porto, Porto, Portugal; ^4^ Institute of Pathology, Leipzig University, Leipzig, Germany; ^5^ Department of Respiratory Medicine, Leipzig University, Leipzig, Germany

**Keywords:** non-small cell lung cancer, lung adenocarcinoma, *KRAS*, co-mutations, resistance to therapy

## Abstract

Lung cancer remains the leading cause of cancer death globally. More than 50% of new cases are diagnosed in an advanced or metastatic stage, thus contributing to the poor survival of such patients. Mutations in the *KRAS* (Kirsten rat sarcoma virus) gene occur in nearly a third of lung adenocarcinoma and have for decades been deemed an ‘undruggable’ target. Yet, in recent years, a growing number of small molecules, such as the GTPase inhibitors, has been investigated in clinical trials of lung cancer patients harboring *KRAS* mutations, yielding promising results with improved outcomes. Currently, there are only two approved targeted therapies (adagrasib and sotorasib) for advanced or metastatic *KRAS*-mutated NSCLC from the second-line setting onwards. In this narrative review, we will focus on *KRAS*, its molecular basis, the role of its co-mutations, clinical evidence for its inhibition, putative mutation to resistance, and future strategies to overcome resistance to KRAS inhibition.

## 1 Introduction

Lung cancer is the leading cause of cancer-related mortality worldwide (1). The poor survival rate of lung cancer patients is mainly due to the late stage of disease found in over half of them at the time of diagnosis (2). Therapeutic progress has been achieved in non-small cell lung cancer (NSCLC) through the introduction of immune checkpoint inhibitors (ICI) (3) and personalized treatment strategies against driver mutations within the tumor, including targeted therapy (4). These driver or oncogenic mutations are localized within kinase domains of receptor tyrosine kinases (RTKs) (5) and are not equally distributed among histologic subtypes of NSCLC (6). Most notably, lung adenocarcinoma (LUAD) harbors those driver mutations and rearrangements that can be therapeutically addressed, such as *EGFR*, *BRAF*, *ALK*, *ROS1*, *RET*, *NTRK*, and also *KRAS* (6, 7). Mutations in the *KRAS* (Kirsten rat sarcoma virus) gene occur in approximately 29–32% of LUAD and, until recently, have been considered to be ‘undruggable’ for the past several decades (8–10).

In the last few years, an increasing number of small-molecule anti-cancer drugs, the so-called GTPase inhibitors as well as others, has been tested in clinical trials, generating encouraging results with improved efficacy of lung cancer treatment for *KRAS*-mutated NSCLC. Presently, sotorasib and adagrasib are the only approved targeted therapies in locally advanced or metastatic *KRAS*-mutated NSCLC patients, but just in those who have received at least one prior systemic therapy. In this narrative review, we will focus on *KRAS*, its molecular basis, the role of its co-mutations, clinical evidence for its inhibition, putative mutation to resistance, and future strategies to overcome resistance to KRAS inhibition.

## 2 Molecular basis of *KRAS* as an oncogenic driver in lung cancer

**Figure 1:**
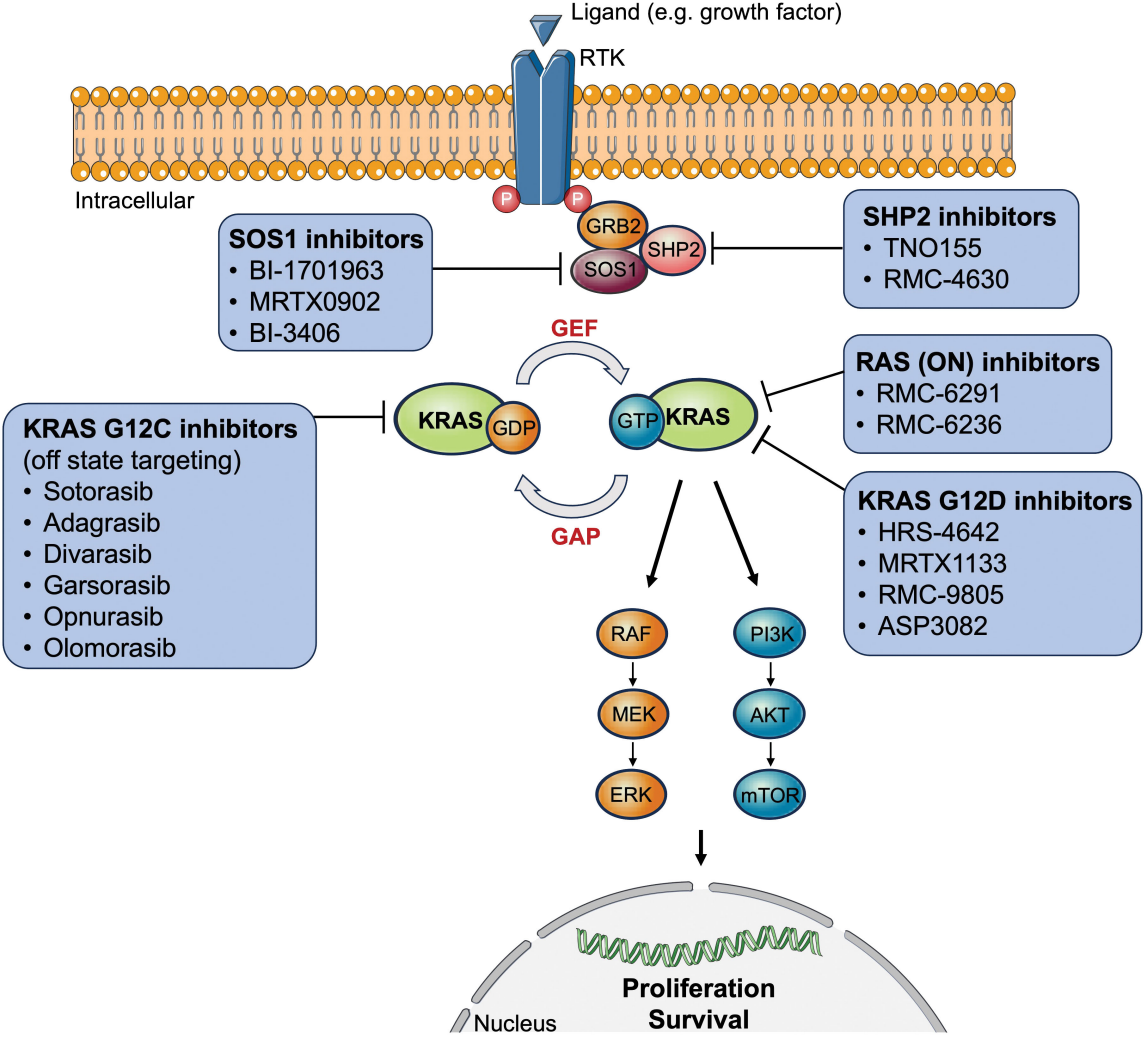
Overview of approved or clinically tested (direct/indirect) KRAS-targeted therapy inhibitors. AKT, protein kinase B; ERK, extracellular signal-regulated kinase; GAP, GTPase activating proteins; GDP, guanosine diphosphate; GEF, guanine nucleotide exchange factor; GRB2, growth factor receptor-bound protein 2; GTP, guanosine triphosphate; KRAS, Kirsten rat sarcoma virus; MEK, mitogen-activated protein kinase kinase; mTOR, mechanistic target of rapamycin; P, phosphorylated tyrosine residues; PI3K, phosphoinositide 3-kinases; RAF, rapidly accelerated fibrosarcoma; RTK, receptor tyrosine kinase; SOS1, son of sevenless 1; SHP2, Src homology region 2 domain-containing phosphatase-2.

The RAS proto-oncogenes encode intracellular guanine nucleotide binding proteins that belong to the GTPase family harboring a catalytic domain and a hypervariable region (11). The former binds guanine nucleotides and activates signaling while the latter determines how RAS proteins are localized on the cell membrane (11). RAS GTPases control downstream signaling by switching between the active nucleotide guanosine triphosphate (GTP)-bound and inactive nucleotide guanosine diphosphate (GDP)-bound states in response to extracellular signals (11). RAS-GTP commonly activates multiple signaling cascades including the canonical RAS-RAF-MEK-ERK (= mitogen-activated protein kinase, [MAPK]), PI3K-AKT-mTOR, and RAS-like (RAL and tumor invasion and metastasis-inducing protein 1 [TIAM1-RAC1]) pathways (11, 12). The first two signaling pathways are most relevant to tumor biology since they play an essential role in cell cycle regulation, thus cell proliferation, and tumor cell survival (**Figure 1**).

In contrast to colorectal cancer and pancreatic adenocarcinoma, the point mutation G12C is the most prevalent genetic alteration in the *KRAS* gene of LUAD, occurring in 39% of cases, followed by the point mutations G12V (18.1%), G12D (13.8%), and G12A (7.2%) (13). However, to date, *KRAS* G12C is the only molecular target for which the two therapeutic agents, sotorasib and adagrasib, have been approved in NSCLC. Conversely, 61% of all *KRAS* point mutations in LUAD are still ineligible for targeted therapy.

## 3 Role of co-mutations with *KRAS*

It is well known that *KRAS* altered NSCLC frequently shows co-occurring mutations with other genes, including tumor protein 53 (*TP53*), serine/threonine kinase 11 (*STK11*), and Kelch-like ECH-associated protein 1 (*KEAP1*), also known as liver kinase B1 (*LKB1*), as well as concurrent amplifications in the *MET* and erb-b2 RTK 2 (*ERBB2*) genes (9, 14–18). Both the triple (*KRAS + KEAP1 + STK11*) and quadruple (*KRAS + KEAP1 + STK11* + *TP53*) co-mutations have been shown to serve as a negative prognostic and predictive factor compared to the single *KRAS* mutational status (15, 18).

Chapter 9 will further elaborate on co-mutations in the context of mechanisms of resistance to KRAS inhibition.

## 4 Clinical evidence for KRAS inhibition in *KRAS*-mutant NSCLC

Historically considered undruggable, *KRAS*-mutant NSCLC now has two approved targeted therapies as well as other potential therapeutic agents that are still under clinical development (10, 13, 19–22). This recent milestone in modern medicine was achieved thanks to the discovery of the allosteric regulatory site of KRAS G12C, thereby leading to the design of irreversible covalent inhibitors (23). Such small compounds bind to the switch-II binding pocket of KRAS G12C (24). Previous crystallography studies were paramount in finding molecules capable of interacting with the unique conformation of the KRAS protein (25). A major scientific breakthrough was made in 2013 with the identification of the switch-II pocket of KRAS by the Shokat Lab, resulting in the structure-based validation of direct targeting of the compound binding region of KRAS in a “mutant-specific” and selective manner (26). The stage was set for the optimization of compounds, leading to the creation of the current KRAS G12C inhibitors available for clinical use today (27). By binding specifically to the inactive GDP-bound form of the KRAS oncoprotein in its switch-II pocket, a covalent bond is created with the mutant cysteine residue of KRAS G12C, blocking the reactivation of KRAS by nucleotide exchange (from GDP to GTP) (27, 28). Hence, KRAS G12C inhibitors essentially trap KRAS G12C in an inactive KRAS-GDP state (off state), hindering a switch to the active KRAS-GTP state (on state), and, thereby, impeding oncogenic activity. This has led to improved drug efficacy and selectivity (29). Currently, sotorasib and adagrasib are recommended by the National Comprehensive Cancer Network (NCCN) guidelines as a subsequent treatment option for patients with *KRAS* G12C-mutant NSCLC in second-line or beyond, if no previous KRAS G12C-targeted therapy was given (30). Given their similar mechanism of action, it is not recommended to switch between these two therapeutic agents at the time of progression (30). **Table 1** summarizes the efficacy data of KRAS G12C inhibitors from published clinical trials.

**Table 1:** Published clinical trials for KRAS G12C inhibitors.

Inhibitor	Study name, phase	Line of treatment	# of patients^2^	Control	ORR^2^ (%)	PFS^2^ (median months, HR)	OS^2^ (median months, HR)	Ref.
Sotorasib	CodeBreaK100, Phase 1	≥2	59	None	32.2	6.3	NA	(31)
	CodeBreaK100, Phase 2	≥2	124	None	37.1	6.8	12.5	(32)
	CodeBreaK200, Phase 3	≥2	171 vs. 174	Docetaxel	28.1 vs. 13.2	5.6 vs. 4.5, 0.66 (P=0.0017)^1^	10.6 vs. 11.3, 1.01 (P=0.53)^1^	(33)
Adagrasib	KRYSTAL-1 Phase 1/2	≥2	116	None	42.9	6.5	12.6	(34) (35),
Divarasib^3^	GO42144, Phase 1	≥2	58	None	60.3	13.1	NR	(36)
Garsorasib	Phase 1	≥2	74 (all doses) 62 (RP2D)	None	40.5 38.7	8.2 7.6	NA NA	(37)

^1^One-sided P-value, ^2^KRAS inhibitor versus control, ^3^neither approved by FDA nor EMA, #, number; ORR, objective response rate (number of patients with complete response plus partial response); HR, hazard ratio; NA, not available; NR, not reached; PFS, progression-free survival; Ref., reference; RP2D, recommended phase 2 dose; OS, overall survival; vs., versus.

The following chapters will give an overview of direct inhibitors of *KRAS* G12C in NSCLC.

## 5 Sotorasib (AMG 510)

The first drug to enter clinical trials geared toward targeting mutant *KRAS*, sotorasib (previously known as AMG 510), was granted accelerated approval by the U.S. Food and Drug Administration on May 28, 2021, for adult patients with previously treated (immunotherapy and/or chemotherapy) locally advanced or metastatic NSCLC harboring the *KRAS* p.G12C mutation (38, 39). In turn, Health Canada approved this KRAS G12C inhibitor in September 2021 (23), while the European Medicine Agency followed suit in January 2022 (Amgen, 2022). These approvals were based on the results of phase 2 of the CodeBreaK 100 trial (32). Preclinical analyses of sotorasib were very promising, showing inhibition of tumor cell growth in both *in vitro* and murine models (40). Sotorasib first entered clinical trial in 2018, and the results of the phase 1 CodeBreaK 100 trial demonstrated encouraging anticancer activity of sotorasib monotherapy in the NSCLC subgroup as follows: 32.2% had an objective response (complete or partial) rate (ORR), 88.1% had disease control (objective response or stable disease), and the median progression-free survival (PFS) was 6.3 months (31). A durable clinical benefit of monotherapy with daily sotorasib (administered orally at a dose of 960 mg) was confirmed in the phase 2 CodeBreaK 100 trial, showing a 37.1% ORR, median PFS of 6.8 months, and median overall survival (OS) of 12.5 months in *KRAS* p.G12C-mutant advanced NSCLC patients previously treated with standard therapies (**Table 1**) (32). The two-year pooled analysis of the CodeBreaK 100 phase 1/2 clinical trial showed that almost 25% of these previously treated advanced stage *KRAS* G12C-mutant NSCLC patients derived long-term benefit from additional sotorasib treatment, with few late-onset treatment-related toxicities (41). These results support the continuing clinical use of sotorasib both in the current therapeutic setting and in studies (ongoing and future) examining its potential role in earlier lines of therapy (41).

In the CodeBreaK 200 study, a randomized, open-label, phase 3 trial (June 2020 to April 2021) of sotorasib (n=171) versus docetaxel (n=174) in the second-line setting and beyond of advanced NSCLC patients with *KRAS* G12C mutation, sotorasib significantly increased PFS (i.e., median PFS 5.6 months [95% CI, 4.3–7.8] vs. 4.5 months [3.0–5.7]; hazard ratio 0.66 [0.51–0.86]; p=0.0017) and exhibited a better safety profile (33). In addition, sotorasib elicited a more rapid (1.4 months vs. 2.8 months) and longer response (8.36 months vs. 6.8 months) compared with docetaxel (33). Unfortunately, although PFS, ORR, and disease control rate (DCR) were improved in the sotorasib group, these results were disappointing when compared to the phase 1 and 2 CodeBreaK 100 trials that showed a longer PFS (6.3 and 6.8 months, respectively) and had a similar ORR and DCR (42).

In addition to sotorasib monotherapy, ongoing clinical studies are also investigating sotorasib-based combinations for the possible treatment of pretreated *KRAS* G12C-mutant NSCLC (20). The single-arm, phase-2 SCARLET study enrolled 30 patients with chemotherapy-naïve, advanced non-squamous, *KRAS* G12C-mutant NSCLC between October 2021 and July 2022 (43). Results from this clinical trial were recently presented in June 2023 at the American Society for Clinical Oncology (ASCO) Annual Meeting, and showed a favorable ORR (88.9%; 80% CI, 78.5–94.8%) (n=27) and tolerability (n=29) for sotorasib plus platinum-doublet chemotherapy (carboplatin/pemetrexed). The PFS and OS rates at 6 months were 61.2% and 87.0%, respectively; median PFS was not reached given the shorter follow-up period (median 4.2 months).

Most recently, exciting positive data from the study arm of sotorasib in combination with carboplatin and pemetrexed for *KRAS* G12C-mutant advanced NSCLC in the ongoing, phase 1b, CodeBreaK 101, global clinical trial have further endorsed the approach to repositioning sotorasib with novel therapeutic combinations into earlier lines of therapy within the treatment paradigm (44). These highly anticipated results, based on a median follow-up of 3 months, were presented at the 2023 International Association for the Study of Lung Cancer (IASLC) World Conference on Lung Cancer (2023) on September 10th, 2023, in Singapore. Patients (n=20) treated in the frontline (i.e., first-line) setting experienced a better ORR and DCR than their counterparts (n=13) treated in the second-line setting (ORR 65% vs. 54%, respectively; and DCR 100%; 95% CI: 83.2, 100, vs. 85%; 95% CI: 54.6, 98.1, respectively). Similar ORRs were reported among patients with programmed cell death ligand-1 (PD-L1) expression less than 1% (i.e., 62% vs. 50% in the frontline vs. second-line setting, respectively). Mature PFS and OS data were unavailable. Due to the very promising results from the global CodeBreaK 101 trial, a new multicenter, randomized, open-label, phase 3 study (CodeBreaK 202) of sotorasib plus carboplatin and pemetrexed as frontline therapy of PD-L1 negative, *KRAS* G12C-mutant advanced NSCLC has been recently initiated by Amgen and is currently recruiting patients (enrollment start date: November 26, 2023; estimated study completion date: March 1, 2031) (45).

## 6 Adagrasib (MRTX849)

Adagrasib is the second approved, orally administered, potent, covalent KRAS G12C inhibitor that selectively and irreversibly binds the switch-II pocket of KRAS G12C (46). Adagrasib was granted accelerated approval by the FDA in December 2022 as a targeted treatment option for locally advanced or metastatic NSCLC with a *KRAS* G12C mutation (47). This decision was based on the results of the ongoing phase 1/2 KRYSTAL-1 clinical trial (**Table 1**) (34, 35). This multicenter single-arm study included patients with histologically confirmed unresectable or metastatic *KRAS* G12C-mutant NSCLC whose disease progressed with frontline chemotherapy and/or immunotherapy. With respect to efficacy outcome measures, 42.9% (95% confidence interval [CI], 33.5 to 52.6) of the 112 patients with measurable disease at baseline had a confirmed objective response. The median duration of response (DOR) was 8.5 months (95% CI, 6.2 to 13.8) and the median PFS was 6.5 months. Confirmed ORRs were similar across PD-L1 expression subgroups (41.7 to 46.8%). The ORRs in patients with co-mutations in *STK11*, *KEAP1*, *TP53*, and *CDKN2A* ranged from 28.6% (*KEAP1*) to 58.3% (*CDKN2A*). As of January 15, 2022 (median follow-up, 15.6 months), the median OS was 12.6 months (95% CI, 9.2 to 19.2).

Updated, longer follow-up data from the KRYSTAL-1 trial, recently presented on September 10, 2023, at the World Congress on Lung Cancer 2023 (WCLC 2023) in Singapore, confirmed durable clinical activity and benefit of adagrasib in advanced *KRAS* G12C-mutant NSCLC across patient groups, including those with CNS metastases and co-mutations (48). Gadgeel and colleagues presented favorable safety and efficacy data (ORR, DOR, PFS, and OS) from a two-year follow-up pooled analysis of the Phase 1/1b Cohort and Phase 2 Cohort A of KRYSTAL-1. As of January 1, 2023, 132 patients received adagrasib, and showed an ORR of 43.0%, with a median DOR of 12.4 months. The median PFS was 6.9 months (95% CI 5.4–8.7), and the median OS was 14.1 months (95% CI 9.2–18.7). Approximately one in three patients (31.3%) remained alive at two years. Exploratory analyses suggested heterogeneity of clinical benefit based on the presence of co-mutations, requiring further evaluation. The safety profile was consistent with previous reports. A confirmatory, multi-center, randomized Phase 3 study, KRYSTAL-12, evaluating adagrasib monotherapy versus docetaxel in patients with previously treated advanced *KRAS* G12C-mutant NSCLC, is ongoing (**Table 2**) (51).

**Table 2:** Ongoing phase 3 trials targeting KRAS G12C.

Inhibitor	Study name, Clinical trial identifier	Combination class	Test arm	Control arm	# of patients	Line of treatment	ORR (%)	DCR (%)	Ref.
Sotorasib	CodeBreaK 202, NCT05920356	Chemotherapy	Carboplatin, pemetrexed, sotorasib	Carboplatin, pemetrexed, pembrolizumab	750	1	NA	NA	(49), no data reported so far
Adagrasib	KRYSTAL-7, NCT04613596	PD-1	Pembrolizumab (PD-1≥50%), adagrasib	Pembrolizumab (PD-1≥50%)	51	1	62.7	84.0	(50)
	KRYSTAL-12, NCT04685135		Adagrasib	Docetaxel	450	≥2	NA	NA	(51), no data reported so far
Opnurasib	KontRASt-02, NCT05132075		Opnurasib	Docetaxel	360	≥2	NA	NA	(52), no data reported so far
Olomorasib	SUNRAY-01, NCT06119581	PD-1 Chemotherapy A: PD-L1 ≥50% B: PD-L1 0–100%	A: Olomorasib, pembrolizumab B: Olomorasib, platinum, pemetrexed, pembrolizumab	A: pembrolizumab B: platinum, pemetrexed, pembrolizumab	1,016	1	NA	NA	No data reported so far

#, number; ORR, objective response rate (number of patients with complete response plus partial response); NA, not available; NR, not reached; PD-1, programmed cell death protein 1; PD-L1, programmed cell death 1 ligand 1, Ref., reference.

It is important to mention that preliminary pharmacodynamics and mechanistic biomarker analysis on pre- and post-treatment tumor NSCLC biopsies of patients (n=3) treated with adagrasib (phase 1/1b and 2) demonstrated down-regulation of *KRAS*/MAPK pathway genes, including *DUSP6* and *SPRY4* (53). Patients with *STK11*-co-mutations had an impressive ORR of 64%. This was a surprising finding given that *STK11* mutations typically portend a poor response and survival to immune checkpoint inhibitors in metastatic NSCLC (54). However, Riely et al. (2021) showed that treatment with adagrasib increased the expression of immune transcripts (e.g., CD4 and CD8) that are minimal at baseline, suggesting a potential immune response to therapy (53).

As noted by Cheema and colleagues (2022), data from preclinical and clinical studies have revealed that drug resistance to single-agent KRAS G12C-targeted therapy occurs quite early after treatment initiation (often within a few months) (23). This suggests that the use of KRAS G12C-targeted therapies in combination with other treatments may help overcome drug resistance observed with anti-G12C monotherapies. Updated, late-breaking data (safety and efficacy results) from the phase 2 KRYSTAL-7 study were recently presented at the European Society of Medical Oncology (ESMO) Congress 2023 in Madrid, Spain (October 20–24, 2023) (55). The results of the KRYSTAL-7 trial, with three patient cohorts stratified according to PD-L1 tumor proportion score (TPS), found that concurrent adagrasib and pembrolizumab in patients with treatment-naïve, advanced, unresectable, or metastatic NSCLC harboring *KRAS* G12C mutation demonstrated encouraging preliminary efficacy with clinically meaningful antitumor activity, especially in patients with high PD-L1 expression (TPS ≥ 50%), and a manageable safety profile (**Table 2**). The patients in this cohort (PD-L1 TPS ≥ 50%) had an ORR of 63% (32/51; 95% CI, 48–76) and a DCR of 84% (43/51; 95% CI, 12.6-not evaluable [NE]). This ORR for the adagrasib-pembrolizumab combination compares favorably with the ORR of pembrolizumab as a single agent (range: 39% to 45%). The median follow-up was longer for patients with PDL-1 TPS ≥ 50% versus all patients (10.1 months vs. 8.7 months, respectively). The median time to response was 1.4 months, and the median PFS was not reached (95% CI, 8.2-NE).

## 7 Intracranial responses with the selective KRAS-G12C inhibitors sotorasib and adagrasib

Patients with *KRAS* G12C-mutant NSCLC are prone to developing brain metastases (BMs) (56, 57). At diagnosis, BMs were detected in 27% to 42% of patients (56, 58–61). *KRAS*-mutant NSCLC patients with untreated central nervous system (CNS) metastases have poorer clinical outcomes (i.e., worse prognosis and higher CNS failure) compared to those without *KRAS* mutations (62–64). For this very important reason, the efficacy of selective G12C inhibitors in the CNS and untreated intracranial lesions remains the subject of intense active research (65). It should be noted that the initial KRYSTAL-1 and CodeBreak100 trials excluded patients with active, untreated BMs (66).

Despite their similarities as allele-specific inhibitors and covalent drugs, sotorasib and adagrasib are indeed different in many ways, reflecting the speed of drug development and their intrinsic properties (67). Notably, with respect to BMs in *KRAS* G12C-mutant NSCLC patients, efficacy data for adagrasib have become available earlier than for sotorasib. Preclinically, adagrasib has shown CNS penetration and its efficacy on *KRAS* G12C-BM in a LU99Luc mouse model showed CNS tumor regression with dose-dependent effects (56). Clinically, it has demonstrated cerebrospinal fluid penetration and BM regression in preliminary findings from the phase 1b portion of the KRYSTAL-1 trial; a retrospective database analysis was initially performed to better understand the clinicopathological features of *KRAS* G12C-mutant NSCLC patients with BM (56). The registrational phase 2 cohort of the KRYSTAL-1 reported findings consistent with the earlier preclinical models of tumor shrinkage, demonstrating an intracranial ORR of 33.3% (11/33 patients) with one intracranial complete response and a median duration of intracranial response of 11.2 months (35). Furthermore, Negrao and colleagues (2023) recently published the first prospective data for the KRAS G12C inhibitor adagrasib in patients with NSCLC and radiologically evaluable, active, and untreated CNS metastases (57). The results of this phase 1b limited BM expansion cohort of the KRYSTAL-1 trial provided proof-of-concept for adagrasib’s ability to penetrate the CNS and achieve promising intracranial activity, with a high concordance rate between intracranial and systemic activity (79%) and a low rate of CNS failure (37%). In early 2024, a case series taken from the KRYSTAL-1 CNS metastases cohort showed that most patients did not discontinue adagrasib because of CNS progression, which was consistent with the overall KRYSTAL-1 CNS metastases cohort and indicated that adagrasib may delay development of additional CNS metastases (68).

Until very recently, published CNS activity data for sotorasib remained relatively scant in comparison to adagrasib (65). Thus far, three case reports describe a remarkable intracranial response of previously untreated, active BMs (69–71). Both Koster et al. (2022) and Yeh et al. (2022) documented a rapid intracranial response in less than two months for their patients treated with sotorasib monotherapy following stereotactic body radiotherapy (SBRT) alone vs. postoperative stereotactic radiosurgery to the cranial resection cavity, respectively, and first-line systemic treatment (i.e., immunotherapy with pembrolizumab) (69, 70). Inno et al. (2023) reported the case of a long duration of intracranial response to sotorasib in the second-line setting lasting 16 months in a patient with both pretreated and untreated symptomatic BMs from *KRAS* G12C mutant NSCLC (71). The importance of exploring dose-dependent CNS response, control, and penetration of the selective inhibitor is emphasized by Lu & Husain (2023) in their case report (65). The patient showed intracranial stability for 5 months on the standard dose of second-line sotorasib monotherapy (960 mg daily), but following a reduction of the sotorasib to 480 mg daily as a result of seizures and vasogenic edema (without new BMs) developed new BMs 5 months later (65).

Clearly, further prospective clinical studies are required to fully characterize the intracranial efficacy of both sotorasib and adagrasib as currently approved therapies as well as other selective G12C inhibitors still in development, including divarasib (GDC-6036) and opnurasib (JDQ-443), among others (66).

## 8 Novel direct *KRAS* G12C inhibitors

In addition to sotorasib and adagrasib, several other direct KRAS G12C inhibitors, such as divarasib (GDC-6063), opnurasib (JDQ-443), garsorasib (D-1553), olomorasib (LY3537982), MK-1084, and JAB-21822 are now in clinical development as monotherapy or in combination with other treatments, as discussed in several recently published reviews (**Tables 2**, **3**, **Figure 1**) (10, 13, 20–22, 82–84). A very recent review touches quite comprehensively and thoughtfully on the manifold combinatorial therapeutic strategies in RAS-driven cancers (84).

**Table 3:** Novel agents for KRAS inhibition.

Inhibitor	Clinical trial identifier, study name, phase	Line of treatment	Mechanism	# of patients	Control	ORR (%)	DCR (%)	PFS (median months, HR)	Ref.
KRAS G12C inhibitor									
Olomorasib	NCT04956640, Phase 1	≥1	Off state inhibitor	KRAS G12C inhibitor naïve, N = 5	None	60.0	80.0	NA	(72)
				KRAS G12C inhibitor treated, N = 9	None	0.0	67.0	NA	
Opnurasib	NCT04699188, KontRASt-01, Phase 1/2	≥2	Off state inhibitor	24	None	42.0	93.0	NA	(73)
IBI351	NCT05005234, NCT05497336, Phase 2	≥2	Off state inhibitor	116	None	46.6	90.5	8.3	(74, 75)
RMC-6291	NCT05462717, Phase 1	≥2	On state, tri-complex inhibitor	KRAS G12C inhibitor naïve (N = 7)	None	42.8	100.0	NA	(76)
				KRAS G12C inhibitor treated (N = 10)	None	50.0	100.0	NA	
MK-1084	NCT05067283, Phase 1	≥2	Unknown	Arm 1: previously treated, receiving MK-1084 monotherapy	None	19.0	NA	NA	(77)
				Arm 2: treatment-naïve, receiving MK-1084 + pembrolizumab	None	47.0	NA	NA	
Glecirasib (JAB-21822)	NCT05009329, Phase 1	≥2	Off state inhibitor	22	None	70.0	100.0	NA	(78)
KRAS G12D inhibitor									
HRS-4642	NCT05533463, Phase 1	≥2	Unknown	10	None	10.0	90.0	NA	(79)
MRTX1133	NCT05737706, Phase 1/2	≥2	Off state inhibitor	NA	None	NA	NA	NA	NA
RMC-9805	NCT06040541, Phase 1	≥2	On state tri-complex inhibitor	NA	None	NA	NA	NA	(80)
Pan/multi-RAS inhibitors (KRAS G12X)									
RMC-6236	NCT05379985, Phase 1	≥2	RAS-multi, on state, tri-complex inhibitor	11 4 with efficacy assessment	None	75.0	100.0	NA	(81)

#, number; DCR, disease control rate (number of patients with partial response or stable disease); HR, hazard ratio; NA, not available; NR, not reached; ORR, objective response rate (number of patients with complete or partial response); PFS, progression-free survival; Ref., reference; OS, overall survival.

Two formerly promising, orally available, investigational, small molecules, LY3499446 and JNJ-74699157 (ARS-3248), were abruptly removed from the G12C inhibitor landscape (82, 83). The discontinuation of the initial phase 1 trial of LY3499446 was due to unexpected toxicity (20, 27). Likewise, JNJ-74699157 (ARS-3248) was investigated in a phase 1 study of patients with advanced solid tumors, including NSCLC (n=5), but enrolment was terminated at just 10 patients due to dose-limiting skeletal muscle toxicities and the lack of efficacy at the lowest administered dose (100 mg) (83, 85).

Data from preclinical and *in vitro* studies have suggested that divarasib (GDC-6063) is more potent and selective than sotorasib or adagrasib (86). In a phase 1 clinical trial, among the 60 NSCLC patients who received divarasib, a confirmed response was observed in 53.4% of patients (95% confidence interval [CI], 39.9 to 66.7), and the median PFS was 13.1 months (95% CI, 8.8 to NE), with an acceptable safety profile (mainly low-grade adverse events) (36).

Opnurasib (JDQ-443), structurally unique and currently in clinical development, has been optimized by design to overcome resistance mechanisms through novel interactions with the binding pocket (83, 87–89). A stable atropisomer with PK/PD activity *in vivo* and dose-dependent antitumor activity in mouse xenograft models, opnurasib has performed in an encouraging manner as evidenced by the early phase data reported from an ongoing Phase 1b/2 clinical trial, with a confirmed ORR of 41.7% (83, 88, 89). As a promising therapy, opnurasib is being investigated in the combination arms of the ongoing, phase 1b/2, multicenter, KontRaSt-01 study, with either TNO155 (SHP2 inhibitor) or tislelizumab (anti-PD-1 monoclonal antibody), as well as in a phase 3 trial of opnurasib monotherapy versus docetaxel (**Table 2**) (73, 83, 90). An update of the KontRaSt-01 was recently presented at the ASCO 2023 Congress, demonstrating promising efficacy and well-tolerated safety data (73).

Garsorasib (D-1553), a novel small molecule inhibitor that selectively targets *KRAS* G12C, is currently in phase 2 clinical trials (91). Preclinical data have already demonstrated antitumor activity of garsorasib. In the phase 1, garsorasib dose-escalation study in *KRAS* G12C-mutant NSCLC patients (n=62), partial response occurred in 24 patients (ORR, 38.7%) and stable disease in 32 patients (DCR, 90.3%) (37).

Olomorasib (LY3537982) monotherapy was tested in a phase-1 clinical trial, in which 5 treatment-naïve and 9 previously treated patients with *KRAS* G12C mutational status showed an ORR of 60% or 0%, respectively, and a DCR of 80% or 67%, respectively (72). The phase-3 SUNRAY-01 trial (NCT06119581) will assess the efficacy of olomorasib in combination with pembrolizumab or pembrolizumab with chemotherapy in 1,016 patients with locally advanced or metastatic NSCLC.

MK-1084 is being tested for *KRAS* G12C mutations as monotherapy in pretreated patients with advanced solid tumors (arm 1) and in combination with pembrolizumab in previously untreated metastatic NSCLC with PD-L1 TPS≥1% in an ongoing, phase 1, global, dose-escalation trial (arm 2) (23). The preliminary results, presented at the ESMO Congress 2023 in October 2023, showed manageable safety and preliminary antitumor activity in both arms (ORR 19% and 47% in arm 1 and 2, respectively) (77).

JAB-21822, now designated glecirasib, was tested in a first-in-human clinical trial comprising 22 patients with advanced NSCLC. The results proved quite promising showing that ORR and DCR were 70% and 100%, respectively (78). Results from future clinical trials are awaited.

## 9 Mechanisms of resistance to KRAS inhibition

The vast majority of advanced NSCLC will progress due to treatment resistance. Tumor cell intrinsic mechanisms are the primary drivers of resistance to radiation, cytotoxic agents, and targeted therapies (6).

Resistance mechanisms to KRAS G12C inhibition cover primary resistance and acquired resistance (92, 93).

Primary resistance or early disease progression (PFS < 3 months) to KRAS G12C inhibitors occurs in about 36% of patients who received sotorasib therapy, as shown in recently published data from the 2-year analysis of the CodeBreaK100 study in NSCLC (41). In NSCLC, co-mutations with genetic alterations in *KEAP1*, *SMARCA4* (SWI/SNF related, matrix associated, actin dependent regulator of chromatin, subfamily A, member 4), and *CDKN2A* (cyclin dependent kinase inhibitor 2A) are associated with inferior clinical outcomes to sotorasib therapy (94). Some studies have demonstrated that co-mutations in *STK11*, *KEAP1*, and *TP53* could modulate the responsiveness of patients with *KRAS* alterations to either KRAS G12C inhibitors or to immunotherapy (14–16, 18, 95). Proulx-Rocray and colleagues (2021) showed that the presence of *STK11* and/or *KEAP1* mutations was associated with a negative impact on survival when compared with wild-type NSCLC patients treated with immune check point inhibitors (96). These authors also reported that in patients harboring *KRAS* mutation, improved prognosis was observed in *STK11+KEAP1* wild-type tumors but not in *STK11+/-KEAP1* mutant tumors. Interestingly, the presence of *KRAS* G12D is associated with diminished infiltration of CD8+ T cells in NSCLC (97). Patients harboring *KRAS* G12D mutations had worse clinical outcomes to PD-(L)1 inhibition compared to wild-type (97). The biological mechanism of resistance mediated by these mutations has yet to be explored. Co-occurring mutations that predict response to treatment might serve as markers for patient stratification and therapy intensification in randomized clinical trials (10).

In terms of allele amplification, high-level amplifications of the *KRAS* G12C allele were observed in some patients undergoing sotorasib treatment (98, 99).

Acquired resistance inevitably occurs and is responsible for disease progression after an initial benefit from targeted therapies. Principly, acquired resistance to KRAS G12C inhibitors are functionally divided into off-target and on-target mechanisms.

On-target resistance mechanisms include alterations that concern the molecular target, against which the inhibitor is directed, such as KRAS. These mechanisms comprise (92, 98, 100):

- Novel *KRAS* mutations in the switch II pocket (e.g. sotorasib: Y96c/d/s, R68S, adagrasib: H95D/Q/R);
- Acquired *KRAS* activating mutation (e.g. G12D on trans and G12W on cis, preventing inhibitor to bind);
- New production of KRAS G12C, and
- *KRAS* G12C gene amplification.

On-target resistance mechanisms were described in a recent *in vitro* study showing that secondary *KRAS* mutations (Y96D, A59T, A59S, R68M, R68M, M721, V8E, G13D, Q61L, Q99L, and H358) conferred resistance to the KRAS (G12C) inhibitors. Moreover, Y96D and Y96S secondary mutations caused resistance to both sotorasib and adagrasib, while the *KRAS* mutations G13D, R68M, A59S, and A59T were highly resistant only to sotorasib and Q99L was resistant to adagrasib but sensitive to sotorasib (101). These acquired mutations were also observed in a clinical study that included *KRAS* G12C-mutant cancer patients treated with adagrasib in monotherapy, of whom 71% were NSCLC patients (98). Furthermore, cell lines with co-mutations of *KRAS* G12C and G12V were described as acquired mechanisms of resistance to KRAS G12C inhibition *in vitro* (102). Similarly, a preclinical and clinical study from Tanaka and colleagues described two *KRAS* activating mutations (G12D, G12V) and a Y96D mutation affecting the cryptic Switch II pocket as mechanisms of resistance during adagrasib treatment (103). Interestingly, G12D-mutant cell lines are reported to have high levels of phosphorylated AKT, leading to the activation of the PI3K-AKT-mTOR pathway (102).

Off-target resistance mechanisms include alterations that comprise upstream and downstream signaling pathways of KRAS as well as histological transformation. These mechanisms comprise (92, 98, 100):

- Activating wild-type isoforms of RAS-proteins, such as NRAS and HRAS;
- Gain of function in oncogenes (e.g. downstream as in the MAPK pathway: *NRAS*, *BRAF*, *MEK1*, *RET* etc.);
- Loss of function in tumor suppressor genes (e.g. cell-cycle transition: *CDKN2A*);
- Gene amplifications, such as in *cMET;*
- Fusion of gene, such as *ALK, RET, RAF1, BRAF, FGFR3*, appear to be more common in colo-rectal cancer;
- Histological transformation (e.g. LUAD to squamous cell carcinoma).

A recent *in vitro* and *in vivo* study demonstrated that *MET* amplification in *KRAS* G12C was associated with resistance to sotorasib *in vitro* and the introduction of a MET inhibitor restored sensitivity by eliminating RAS–MEK–ERK and AKT signaling (104). Furthermore, MET copy level gain was an off-target mechanism of resistance to sotorasib in a patient with *KRAS* G12C-mutant LUAD (105). Activating mutations in *NRAS*, *BRAF*, *MAP2K1*, and *RET*; oncogenic fusions involving *ALK*, *RET*, *BRAF*, *RAF1*, and *FGFR3*; and loss-of-function mutations in tumor suppressor genes, such as *PTEN* and *NF1*, were described as acquired off-target resistance mechanisms of KRAS G12C inhibitors (19, 92, 101, 106).

**Table 3** and **Figure 1** give an overview of three potential agents targeting *KRAS* G12D mutations: HRS-4642, MRTX1133, and RMC-9805. Moreover, G12V mutations are shown to preferentially activate RAL signaling (102).

## 10 Future strategies to overcome resistance to KRAS inhibition

For NSCLC harboring a *KRAS* G12D mutation, there are several specific inhibitors undergoing testing in clinical and preclinical studies (**Table 3**, **Figure 1**). MRTX1133 is a non-covalent KRAS G12D inhibitor that showed significant preclinical antitumor activity in *KRAS* G12D-bearing tumor cells, especially pancreatic ductal adenocarcinoma (107). This compound might be a potential treatment in combination with KRAS G12C inhibitors for patients harboring co-mutations (*KRAS* G12C, G12D). Further studies are needed to clarify the role of adaptive resistance mechanisms in acquiring resistance to KRAS inhibitors.

RM-018, a tricomplex KRAS G12C active-state inhibitor, retains the ability to inhibit KRAS (G12C, Y96D) (103), thus being a promising therapy to address acquired resistance. Adaptive resistance mechanisms due to reactivation of MAPK pathway and upregulation of PI3K-AKT pathway were identified as likely resistance mechanisms and, according to *in vitro* and *in vivo* models, combination with PI3K inhibitors could overcome this resistance (108).

Several studies have uncovered the mechanisms underlying resistance to KRAS G12C inhibition and there have been pioneering efforts to overcome drug resistance using combinatorial treatments (108–111).

One approach is to target upstream effector proteins of the KRAS protein itself. For instance, the phosphatase son of sevenless homolog 1 (SOS1) is a RAS guanine nucleotide exchange factor (RasGEF), which is activated by SHP2 promoting RAS activation through GTP binding (**Figure 1**) (112). The combination of a novel SOS1 inhibitor (BI-3406) and trametinib exhibited potent activity against Y96D and Y96S (113). In addition, other SOS inhibitors, such as BI-1701963 and MRTX0902, are currently being tested in clinical trials (10).

SHP2 is another upstream adapter protein that is phosphorylated upon activation of RTK. Two SHP2 inhibitors are currently under clinical investigation: TNO155 and RMC-4630 (10, 13). KRAS G12C inhibitors in combination with SHP2 inhibition led to sustained RAS pathway suppression and improved efficacy *in vitro* and *in vivo* (111).

Recently, a phase 3 clinical trial showed that sotorasib in combination with panitumumab (EGFR inhibitor) resulted in longer PFS than standard treatment in metastatic colon cancer patients (114). Further studies are needed to test whether this combination could improve the outcome in lung cancer. Promising evidence has demonstrated that adagrasib plus pembrolizumab improves overall response rate in patients with newly diagnosed NSCLC harboring a *KRAS* G12C mutation, particularly in those with higher levels of PD-L1 (115).

As such, specific therapeutic combinations may help in cases of either intrinsic resistance or acquired resistance.

## 11 Conclusion

The *KRAS* mutation plays a major role in the development of tumor progression and resistance to treatment. Despite this, G12C point mutation (making up only 39% of all KRAS alterations) remains the only molecular target for which the two therapeutic agents, sotorasib and adagrasib, have been approved so far. The advent of novel inhibitors against *KRAS* mutations will further improve survival of lung cancer patients. Nevertheless, the co-occurrence of add-on mutations (co-mutations) and by-pass track pathways will remain challenging obstacles to overcome since they reduce treatment success. Future research efforts must be directed toward comprehensive molecular testing of lung cancer, allowing for the development of multimodal treatment strategies including immune checkpoint inhibitors, tyrosine kinase inhibitors, KRAS upstream inhibitors, and multi-kinase inhibitors against co-mutations.

## Author contributions

KS: Writing – original draft, Writing – review & editing. MC: Writing – original draft, Writing – review & editing. MV: Writing – review & editing. AF: Conceptualization, Data curation, Formal analysis, Funding acquisition, Investigation, Methodology, Project administration, Resources, Software, Supervision, Validation, Visualization, Writing – original draft, Writing – review & editing.
